# Relevance and quality of the Gipuzkoan extracurricular youth sports program: an evaluation from a positive youth development perspective

**DOI:** 10.3389/fpsyg.2024.1404110

**Published:** 2024-09-25

**Authors:** Ekain Zubizarreta, Jon Ortuondo, Oidui Usabiaga, Nagore Martinez-Merino

**Affiliations:** ^1^Department of Physical Education and Sport, Sports Faculty of the University of the Basque Country (UPV/EHU), Vitoria-Gasteiz, Spain; ^2^Teacher Training University College of Begoñako Andra Mari, Bilbao, Spain; ^3^Department of Didactics of Musical, Plastic and Body Expression in the Faculty of Education of the University of the Basque Country (UPV/EHU), Bilbao, Spain

**Keywords:** sport programs, physical activity and sport, positive youth development, program evaluation, extracurricular sports program

## Abstract

Physical activity and sport (PAS) programmes are an important part of organized extracurricular activities for children and young people. They appear as potentially inclusive environments where students could be provided with holistic development based on active and healthy lifestyles. The aim of this study was to evaluate and describe the Gipuzkoa School Sport Programme (GSSP) from the positive youth development (PYD). The research was carried out based on CPAR (Critical Participatory Action-Research) methodology: a “CPAR group” was created -including researchers and policymakers- to carry out the evaluation of the GSSP in which we analyzed (a) the social environment or PYD climate and (b) life skills. The evaluation and description of the GSSP confirmed that it meets most requirements for providing positive development contexts and opportunities for PYD. The choice of prioritizing multisport and deliberate play and putting early specialization aside seem key conditions to ensure PYD-promoting environments. This work could serve as a referent for decision-makers in organizations dedicated to designing, developing, implementing, and evaluating PYD-focused extracurricular youth sport programmes.

## 1 Introduction

Out-of-school time activities take place in broad social and cultural ecology contexts and may hinder or enhance the development of children and adolescents (Vandell et al., 2015). Furthermore, youth development can be conceptualized from a problematic behavior prevention approach—in which the young person is regarded as a “problem”—or it can be viewed through a more contemporary lens that emphasizes opportunity or positive development (Lerner et al., 2011, 2013). As highlighted by Roth et al. (1998), positive development can be considered as “engagement in pro-social behaviors and avoidance of health-compromising and future-threatening behaviors.” It is in this latter positivity-oriented perspective that the positive youth development (PYD) theoretical framework is based. According to it, activities organized outside school hours can provide young people with positive developmental contexts and opportunities to experience PYD (Mahoney et al., 2006; Oberle et al., 2019).

Physical activity and sport (PAS) programmes are an important part of organized out-of-school-time activities for children and young people. According to Holt and Neely (2011, p. 311), providing children with multiple PAS contexts that encourage PYD and supporting them through appropriate policies could help create conditions that enable them to thrive, lead healthy and fulfilling lives, and have meaningful community participation as adults. The Holt et al. (2017) model can serve as a guide that details the characteristics extracurricular PAS should have. This model emphasizes the need to create an appropriate social environment that enables young people to acquire life experiences and skills that will contribute to PYD outcomes.

Scientific evidence has confirmed how regular PAS practice during childhood and adolescence leads to several physical, psychological, and social benefits (Chaput et al., 2020). However, when promoting PAS at school, it is argued that the competent public administrations should not only focus on meeting the World Health Organization’s (World Health Organization, 2020) recommendations for this population group, but should also emphasize issues such as gender equality, equity and social justice, student and family heterogeneity, sustained or lifelong participation, accessibility to sports facilities and sustainable travel, etc (Camiré et al., 2021; Cassar et al., 2019; Evans et al., 2017). If these are considered, out-of-school time PAS presents itself as a potentially inclusive and ideal environment to provide students with holistic development based on active and healthy lifestyles (Gu et al., 2020).

Childhood and adolescence PAS programmes promote several important developmental outcomes for young people, such as the training of future elite athletes, Sport for All, the development of lifelong sport-related and healthy habits, instant fun, or positive development (Côté and Hancock, 2014; Siedentop, 2002; Waldron et al., 2020). However, these studies confirm that youth PAS programmes are largely structured to achieve these outcomes independently. This creates difficulties for policy makers (Coalter, 2010), to the point that it remains a major challenge for them to develop out-of-school time PAS programmes that meet the multiple needs of young participants (Côté and Hancock, 2014). Additionally, another challenge is to decide whether early childhood PAS programmes should be oriented toward diversification or specialization. According to Côté et al. (2009), the many benefits of multisport practice lead to positive child development, which could clearly tip the balance in favor of a multisport option.

However, the purpose of this study was to describe and evaluate the Gipuzkoa School Sport Programme (GSSP) of the Provincial Council of Gipuzkoa (PCG) from the Holt et al. (2017) “PYD through sport” perspective. It should be noted that the present research stems from a request from the policy makers in charge of the GSSP, whose aim is to improve the current policy based on scientific evidence.

### 1.1 The Gipuzkoa School Sports Programme

According to Article 10.36 of the Statute of Autonomy (Basque Government, 1979), the Basque Autonomous Community—located in the north of Spain and made up of the Historical Territories of Araba, Bizkaia and Gipuzkoa—has exclusive competence in the field of sport. Said competence is reflected in Law 14/1998 of 11 June 1998, on Sport in the Basque Country (Basque Government, 1998). Specifically, Decree 125/2008 on School Sport in the Basque Country (Basque Government, 2008) elaborates on school sport. It defines school sport as a sporting activity organized outside school hours and practized by schoolchildren during compulsory education (between ages 6–16). Under this autonomous regulation, the regional bodies of the Historical Territories annually approve the school sport programme, which pursues the following general objectives: (a) to complement the children’s comprehensive school education, and (b) the harmonious development of their personality, (c) the attainment of adequate physical and health conditions and (d) to provide training that enables the continued practice of sport at later stages of life (Basque Government, 2008, p. 4).

In the case of Gipuzkoa,^1^ the E1 report (Provincial Council of Gipuzkoa, 1992), which contains the first structural solution for the GSSP was drawn up and implemented by the PCG Sports Service 30 years ago. The recent E2 report (Provincial Council of Gipuzkoa, 2022) is an updated proposal based on scientific evidence and international recommendations, which represents an evolved structure of an introduction to sports model aimed at placing the educational process above sporting results. Thus, there are eight main objectives that the current GSSP (Provincial Council of Gipuzkoa, 2022) aims to achieve: (a) educating schoolchildren; (b) reaching the entire school population; (c) promoting an active and healthy life; (d) fostering communities and community development; (e) promoting motor activities in Basque language and Basque culture; (f) developing the sport itself; (g) fostering sporting talent, and; (h) guaranteeing children’s rights during the introduction to sport.

According to data regarding school sport enrolment (Provincial Council of Gipuzkoa, 2022), a total of 47,409 people (46% of which were girls) participated in the GSSP in the 2021–22 school year—72% of Primary school pupils and 58% of secondary school pupils.

The organization, financing, and implementation of the GSSP requires the participation and coordination of several agents (Provincial Council of Gipuzkoa, 1992) such as: schools, school parents’ associations, local federations and sports clubs, town councils and the PCG. Federations provide technical assistance for the activities—a public function delegated by the administration—while town councils implement the GSSP in coordination with the Provincial Government, mainly through the stimulation of local school sport structures, facility provision, and programme financing. On the other hand, schools assume the following functions (among others): design of their activity offer in coordination with the local structure, coordination with the rest of nearby schools—in the area, the municipality and even in the rest of the region—for activity development, encouragement of pupil participation, provision of technical staff and execution of the administrative work derived from the activities.

It should be noted that in Gipuzkoa, a series of measures have been established in agreement with the rest of the agents involved in the school sport ecosystem, such as (Provincial Council of Gipuzkoa, 2022): (a) structuring the school as the basic core location for schoolchildren’s participation; (b) defining the minimum age for participation based on the type of activity and pathway and, specifically, the introduction of sporting activities starting the third year of primary education (age 8); (c) limitation of the activities’ geographical scope according to student age; (d) regulation of the competitions and the teaching of the sport modalities; (e) absence of classifications through the fourth year of primary education (age 10), and; (f) adaptation of the sport regulations.

The GSSP consists of three sport pathways (Table 1) which, in turn include several activities and programmes (Provincial Council of Gipuzkoa, 2022): the participation pathway, the introduction to performance pathway and the talent pathway:

**TABLE 1:** Pathways and types of activities in the GSSP, by age.

Pathways	Activities		PS1	PS2	PS3	PS4	PS5	PS6
(A) Participation pathway	Introduction activities	Development of basic motor skills Single sport Multisport Sports meetings						
	
	Participation competitions							
(B1) Introduction to performance pathway	Detection activities							
	Technification activities Introduction to sports performance competitions							
					
(B2) Talent detection pathway	Detection activities							
	Technification activities Restricted competitions							
					

Source: Translated from the GSSP programme (Provincial Council of Gipuzkoa, 2022).

(1) The aim of the participation pathway is for schoolchildren to get to know and practice different sports and to acquire sporting habits. This pathway contains two main activities:

(a) Introduction activities provide the possibility to experience various PAS outside a competitive environment. The starting age is established according to each modality’s characteristics. In most cases, children start in the third year of primary school (age 8), except for those activities that favor basic motor skill development, for which they start in the first year of primary (age 6). This latter group is included in the Jolas Hezi programme (ages 6 and 7) which is implemented within the school (Provincial Council of Gipuzkoa, 2017). The aim of the Jolas Hezi programme is to promote motor development and adherence to physical activity among the school population through games and recreational-motor activities. Currently, 43% of schools in Gipuzkoa offer this programme. Regarding inclusiveness, and in line with the PCG’s disability policies (Provincial Council of Gipuzkoa, 2018), the Regional Government provides financial, material, and advisory resources to guarantee the participation of disabled schoolchildren at the school center and with their peers through the inclusive (ISS) and/or adapted (ASS) school sport programmes (Provincial Council of Gipuzkoa, 2022).
(b) Participation competitions, outside of a purely selective scheme and without any restrictions on the number of participants, aim to provide schoolchildren from the third year of primary school onward the possibility to participate in competitive activities or meetings through their school or sports club. In line with the objectives pursued by the GSSP, schools prioritize the multi-sport offer, known as the Multikirola Programme, offered between the third and sixth years of primary school. Thus, participation through sports clubs can be regarded as a complement and not as a substitute for participation in the Multikirola Programme at this stage. Choosing a sport to specialize in is delayed until the start of secondary school (age 12). Based on the enrolment data of the GSSP application for the 2021–2022 school year, Multikirola is offered in 85% of schools in Gipuzkoa and 57% of the enrolled pupils participate.

(2) The introduction to performance pathway aims to provide interested schoolchildren the opportunity to get started in an objective-focused practice,
(3) The talent detection pathway aims to find, select, and train pupils who show attitude and ability to become promising young athletes.

## 2 Materials and methods

The CPAR methodology (Critical Participatory Action-Research) (Kemmis et al., 2014) was used to address the identified needs, and project objectives were set jointly. To study “social issues” (Fals-Borda, 1979) from a critical perspective, Kemmis et al. (2014) highlighted the importance of both the scientific procedure and the results obtained, which is why collaboration between researchers and the individuals involved is key. Thus, a “CPAR group” (Kemmis et al., 2014) including researchers (4) and policy makers from the PCG (2) was created. This group devised a strategy and proposed a multiple stage “planning, acting, observing, reflecting” cycle process (Kemmis et al., 2014). This article focuses on the first phase of the research, which aims to use evaluation tools developed from a PYD perspective and to analyze the GSSP programme.

For this purpose, Tejedor (2000) guidelines of programme evaluation were used, and the following areas were addressed for the research design:

(a) Establishing the activities or programmes to be evaluated:
The GSSP is organized based on guidelines and regulations of the PCG and the Basque Government. Specifically, the programme’s three main regulatory documents were analyzed: (a) Decree 125/2008 on School Sport of the Basque Country; (b) Provincial Order 40-133/2021 and (c) E1 and E2 reports.
(b) Establishing evaluation criteria:
Since the objective was to evaluate the programme from a PYD perspective, the evaluation criteria were divided according to Holt et al. (2017) into two main groups: “PYD climate” and “Life skills program focus.” The third category proposed by Holt et al. (2017)—“PYD outcomes”—was excluded from the analysis given that this first phase of the study has been limited to programme design and did not include programme implementation.
(c) Choosing information extraction strategies, and (d) information analysis and decision making:
Once the documents had been agreed upon with the research group, external PCG researchers analyzed and coded the documents according to the PYD categories by using content analysis (Rapley, 2014). To guarantee scientific rigor criteria (Lincoln and Guba, 1986) such as trustworthiness and authenticity, each researcher coded the results individually and then analyzed between-peer coding to clarify the differences identified. Once the pairwise process was completed, responses were discussed in the CPAR group to prepare and write up the results.

The present study was approved by the Human Research Ethics Committee (CEISH, cod. M10_2021_252) of the University of the Basque Country (UPV/EHU) and followed the guidelines established in the Declaration of Helsinki (World Medical Association, 2013).

## 3 Results and discussion

### 3.1 Multisport practice and positive youth development

The decree 125/2008 on School Sport of the Basque Country stipulates that “school sport shall preferably be multi-sport,” and the GSSP goes a step further by developing a model of introduction to sport based on and oriented toward multi-sports, thus avoiding early specialization of schoolchildren. Many studies show that multisport practice at early ages produces significant benefits in terms of improving future sports performance (LaPrade et al., 2016), mental healthcare (Giusti et al., 2020), injury prevention (Carder et al., 2020), adherence to physical activity (Roetert et al., 2018) and the development of social skills (DiStefano et al., 2018), among others. Along the same lines, Côté et al. (2009) argue that multisport practice enables children to experience many different physical, cognitive, affective, and psychosocial environments, which has a positive impact on their overall development. Ultimately, the backbone of the GSSP programme –multisport grounded in deliberate play—provides a more conducive context for promoting PYD compared to an early specialization-oriented sports programme (Holt et al., 2017; Waldron et al., 2020).

In the following sections, the GSSP programme is analyzed using the Holt et al. (2017) model as a foundation, with a particular focus on multisport practice. A first section includes the measures taken by the PCG regarding PYD climate, while a second section describes life skill building activities.

### 3.2 Social environment: PYD climate

According to Holt et al. (2017, p. 32), PYD climate is a result of the contextual and social characteristics of a sport activity, which provide young people the opportunity to acquire PYD-contributing experiences. Côté et al. (2009) state that a programme based on multisport can help obtain positive social, physical, and personal results—provided the context in relation to peers, parents and coaches is appropriate. In this regard, and in line with was is suggested by Holt et al. (2017), the GSSP advocates the presence and development of an educational community that promotes: (a) the increase of social cohesion among all the agents involved in school sport; (b) the creation and development of human capital; (c) the reduction of feelings of loneliness and helplessness among participants and; (d) the improvement of the integration processes of migrants or disabled people (Provincial Council of Gipuzkoa, 2022, p. 21). Likewise, practicing sport within this community allows students the opportunity to stay active, and encourages the acquisition of new motor skills, provides room for new friendships, and reduces antisocial and aggressive behavior.

Thus, this dimension has been analyzed following Holt et al. (2017), by subdividing it into three categories: (a) relationship with adults; (b) relationship with peers, and; (c) parental involvement.

#### 3.2.1 Relationship with adults

In order to ensure the harmonious development of schoolchildren and strong, healthy and positive relationships between them and the adults responsible at the GSSP, it is recommended that the latter are adequately qualified (Kavussanu et al., 2008). Therefore, GSSP multisport programme supervisors must at least have professional training as Sports Supervisors, while coaches must at least have completed the first cycle of the Sports Technician Degree in the corresponding modality. In turn, coordinators must have a Degree in Physical Activity and Sport, a Degree in Primary Education with a focus on PE or a higher qualification in the corresponding modality (Provincial Council of Gipuzkoa, 2022, p. 72).

Another significant feature is the existence of a coordinator with pedagogical functions. Thus, aside from conventional management duties, the coordinator is in charge of creating a direct link between the school staff and the sport supervisors in order to ensure pedagogical coherence between the school’s educational project and the school sport activities. Additionally, the coordinator must supervise and guarantee that the sport’s supervisors’ duties are aligned with the pedagogical objectives (Provincial Council of Gipuzkoa, 2022, p. 71).

Regarding the training and pedagogical functions of the adults responsible for the GSSP, it is promoted that these tasks are carried out by qualified personnel from an educational perspective, with the ultimate goal of generating positive effects on the participants (Moreno-Arrebola et al., 2017). According to Loughead and Carron (2004), focusing support on positive, non-autocratic reinforcement leads to better cohesion results among athletes. On the other hand, Contreras (2006) states that sport participation *per se* is not necessarily educational, and that specific measures are needed to ensure that this is the case—this is the intention of the GSSP.

Likewise, people employed in the GSPP enjoy some degree of job stability, and the program encourages a model where managers and leaders of sport activities have an employment contract (Provincial Council of Gipuzkoa, 2022, p. 72). This regularization of labor may favor job stability, which directly influences the programme’s quality and recognition (Rivera et al., 2010). Interestingly, however, some international school sport programmes advocate a model in which supervisors and coaches are volunteers who perform their work solely for motivation, personal growth, or personal satisfaction (Busser and Carruthers, 2010; Edwards and Kulczycki, 2021).

Lastly, child protection is a paramount concern, which is why all technical staff involved in the activities are required to possess a clean certificate of sexual offenses (Provincial Council of Gipuzkoa, 2021, p. 9). These measures are intended to protect schoolchildren from situations of abuse or harassment which, as the literature confirms, are not uncommon (Hartill, 2016; Kerr et al., 2019).

#### 3.2.2 Relationship with peers

In this second subcategory, Holt et al. (2017) address strong relationships between young people and the sense of community belonging which lead to a PYD climate. In this regard, the GSSP frequently and intentionally refers to the “group” as a basis for collective, positive, and enriching experiences. Students are introduced to sports from within the group—and under its protection –, which aims to promote social skills, teamwork, and mutual respect. Furthermore, Multikirola groups are generally “closed” so to foster group cohesion and allow for better monitoring of participant’s learning process, which is developed within the stable group with an assigned supervisor, regardless of the different sports modalities that are practiced (Provincial Council of Gipuzkoa, 2022, p. 60). Social environment and peer involvement influence intrinsic and extrinsic motivation toward PAS (Li et al., 2014) and, accordingly, closed groups were chosen for the GSSP.

As to building strong peer relationships, it should be highlighted how gender perspective is integrated in the GSSP:

“Including a gender perspective is key to ensuring equal participation of students of different sex in the E2 model. Additionally, this provides an opportunity to overcome conflict situations and to put an end to the endless cyclical debate between mixed and segregated proposals. It will also be an invitation to design more effective strategies to increase the participation of girls. An emphasis is put on the need to study the impact of socio-cultural macro and micro variables on the participation of girls and boys rather than on the student’s gender” (Provincial Council of Gipuzkoa, 2022, p. 41).

Furthermore, it clearly states it in the Jolas Hezi programme, which is focused on the motor empowerment of girls, with a particular emphasis on motor skills and methodology (Provincial Council of Gipuzkoa, 2022, p. 54). The GSSP seeks to encourage the participation of girls to improve their motor ability and therefore their tendency to adhere to PAS, as highlighted by Arribas-Galarraga et al. (2018). Given the link between perceived motor competence and sport dropout in adolescence, increasing motor literacy of female students at this age may help prevent them from dropping out of sport (Lopes et al., 2011).

Similarly, the GSSP seems to welcome diversity (Ainscow, 2005), and stresses that admission requirements shall not become “a cause of discrimination on the grounds of birth, race, sex, religion, opinion or any other personal or social condition or circumstance, nor may they be applied arbitrarily or vexatiously” (Provincial Council of Gipuzkoa, 2021, p. 7).

Beyond discrimination on the above-mentioned grounds, which remains an educational and social challenge (Spaaij, 2009), the GSSP strives for inclusivity by materializing this attention to disabled people through the ISS and ASS programmes mentioned above. Thus, though inclusive spaces may not necessarily bring about social interactions (Rodríguez-Rosa and Beckman-Menezes, 2019), the GSSP is committed to creating spaces that can foster different types of inclusion levels (Qvortrup and Qvortrup, 2018) and resembles model of social inclusion (Bailey, 2005).

Aside from the focus on disabilities, the GSSP means to remove socio-economic barriers to school sport by fostering “public resources to facilitate access for people with financial difficulties” (Provincial Council of Gipuzkoa, 2022, p. 37), thus ensuring more equitable and inclusive access (Bailey, 2005).

#### 3.2.3 Parental involvement

This last subcategory addresses family support, which Holt et al. (2017) highlight the importance of for boosting PYD.

On this matter, the GSSP stresses the importance of a close relationship between the people responsible for programme management and the schools’ parents’ associations (Provincial Council of Gipuzkoa, 2022). Article 2.2 of Decree 125/2008 states that the GSSP must be integrated into each school’s educational project and annual plan. Therefore, the PAS offered at schools require the approval of both the teaching staff and the families (Provincial Council of Gipuzkoa, 2022, p. 36). This is aimed at involving the educational community (teachers, families, and students) in the planning, development, monitoring and evaluation process of the GSSP (Provincial Council of Gipuzkoa, 2022, p. 59), which in turn improves consistency between the programme’s and the school’s educational focuses. According to several authors (Strachan et al., 2011; Turnnidge et al., 2012), the involvement of families in sport programmes is crucial to create an educational environment, to the point that it is sometimes suggested that parents are included as part of the team.

According to the GSSP, family engagement is necessary for the activities to become positive, diverse, and enriching experiences (Provincial Council of Gipuzkoa, 2022, p. 22–23). This is due to the direct influence of family on both children’s practice and on whether said practice takes place following educational criteria (Li et al., 2014).

Also, the GSSP considers that parental involvement can potentially enrich between-parent relationships, which has a positive influence on the communities. The programme underlines that school sport may provide an ideal environment for families of diverse backgrounds to mingle, and relationships created during sporting events or sports-related trips enhance community building and have a positive impact on human capital (Provincial Council of Gipuzkoa, 2022, p. 23).

Additionally, the Jolas Hezi programme proposes three family meetings to encourage the involvement of different generations in the extracurricular activities (Provincial Council of Gipuzkoa, 2022, p. 55). Many studies have investigated the influence of intergenerational PAS programmes involving both children and adults. Most of them state that they have positive effects on both generations in terms of improved quality of life, psychological wellbeing, improved attitudes toward others, reduced prejudice and negative attitudes, increased self-esteem, and improved school performance (Buonsenso et al., 2021).

### 3.3 Life skills

No specific information regarding the acquisition and transmission of life skills developed through extracurricular PAS has been found among the GSSP documentation. However, we believe that the GSSP’s educational approach favors schoolchildren’s gain of life skills. The GSSP is developed and implemented in educational centers, as indicated in the following GSSP text:

School contexts are the most effective spaces to promote physical activity for children and youth. This comprehensive approach, along with the E2 school sports program, advocates for more and better Physical Education. It involves organizing active commutes, implementing strategies to reduce sedentary time, providing spaces and opportunities for physical activity outside of school hours, and recommending educational programs that engage families and the entire educational community. Within this comprehensive strategy, with the school as the ideal context to ensure educational sports initiation for all students, the E2 model prioritizes the development of school sports programs within the institution (Provincial Council of Gipuzkoa, 2022, p. 35).

In this regard, school sports programs should be aligned with the educational objectives of the current curriculum and the overarching mission of each school. Thus, it seems as though an atmosphere of child and youth development is generated through the objectives, measures and activities established in this community sports programme. This fosters both positive developmental experiences in young people, in accordance with Fraser-Thomas et al. (2005) and encourages the teaching of skills transferrable to other domains of life—implicitly and explicitly –, as proposed by Holt and Neely (2011). All in all, the GSPP’s philosophy is in line with that of Holt et al. (2017), who argue that programmes aimed at promoting positive youth development would be more effective if they emphasized the acquisition and transmission of life skills and abilities. From this perspective, the main GSSP features associated with the transfer of life skills among participating schoolchildren are explained in the following sections.

The GSSP considers the educational environment as the safest and most appropriate in which to develop life skills. Thus, the learning process is put before the sporting outcome (without despising the introduction to sports performance). The choice of this context is intended to provide the opportunity for the “harmonious and holistic development” (Provincial Council of Gipuzkoa, 2022, p. 13) of the participating schoolchildren. Accordingly, one of the measures aimed at achieving this is to implement the programme outside school hours at the school where the children study—school is considered the most suitable institution, as it makes the interests and needs of young people a priority (Provincial Council of Gipuzkoa, 2022, p. 5, 15). Ultimately, placing the GSSP in an educational context enables the development of life skills from a social justice lens, as explained by Camiré et al. (2021). Furthermore, other of the programme’s measures—such as limiting the geographical scope of activities according to children’s age, regulating competitions and the teaching of sport modalities, or not using classifications until the age of 10—help to promote social justice and thus allow for exploration of other motor practices, cultural forms, identities, and lifestyles that do not replicate modern hyper-competitive, hierarchical, and patriarchal sports (Atkinson, 2010, p. 112). In short, the aim is to lessen the impact of sports clubs’ focus on performance by putting the spotlight on schools and limiting competitive and single-sport practice. Putting educational objectives before competitive ones and prioritizing the less result-oriented and more playful multi-sport practice (Provincial Council of Gipuzkoa, 2022, p. 43–44) encourages the transferability of life skills and learning for all.

Reviewed literature emphasizes that school sport aims to improve participants’ intrinsic motivation (Provincial Council of Gipuzkoa, 2022, p. 32, 43), self-esteem (p. 21) and academic skills, as well as to decrease the likelihood of psychological problems (p. 31, 34). Once again, the role of the school (p. 46) seems to be pivotal for participants to develop mental capacities and skills for life, as it offers a safer context, free from third party influences that promote more result-oriented models (p. 38). As far as the acquisition of a sports habit and other healthy habits, the programme aims to reduce levels of physical inactivity and prevent early drop-out from sport. According to the programme, one of the keys would be to make school sport playful. When introduction to sport happens in suitable contexts, organized activity can become a powerful tool for the healthy development of the infant-young population and provide broad psychosocial benefits (Evans et al., 2017).

The GSSP aims to convey social development of participants. On the one hand, it seeks to develop social and communication skills (Provincial Council of Gipuzkoa, 2022, p. 21, 24). The promotion of multi-sport practice allows—aside from its inherent richness—for the development of intra and interpersonal life skills, the exploration of each person within their own personality and the encouragement of prosocial behaviors (Côté et al., 2009). Exposure to different types of sports helps schoolchildren to adequately develop their social skills (DiStefano et al., 2018). On the other hand, sport is intended as a tool for the development of ethical and civic values such as respect, tolerance, inclusion and a gender and equality perspective (Provincial Council of Gipuzkoa, 2022, p. 42), which prove useful to participants in facing some of today’s challenges (p. 21).

## 4 Conclusion

After the evaluation and description of the GSSP, it is confirmed that it meets many of the requirements for harboring positive development contexts and opportunities for PYD. The PCG’s bid for multisport and deliberate play, with a clear disregard for early specialization, is one of the key features in providing PYD-promoting environments.

The main measures included in the GSSP regarding PYD climate are: (a) guaranteeing the training of the programme staff; (b) ensuring the existence of a coordinator with pedagogical functions; (c) promoting the employment regularization of adults involved in the GSSP; (d) requiring a clean certificate of sexual offenses for all technical staff; (e) creating closed groups throughout the school year; (f) reflecting plurality and diversity in the admission requirements; (g) fostering access of students with financial difficulties, and; (h) encouraging parental involvement.

The GSSP’s educational approach encourages the acquisition and transmission of life skills among school children. In this regard, the following measures may be highlighted: (a) placing educational focus before sporting results; (b) carrying out the extracurricular activities at the same school; (c) limiting geographical scope of the activities in accordance with student’s age; (d) not using classifications until 5th grade of primary, and (e) making school sports playful.

The Holt et al. (2017) model is an interesting tool for evaluation from PYD extracurricular youth sport program perspective. However, we believe that this evaluation might be enriched by considering community transformation, gender perspective, equal participation, diversity, or location-specific cultural and linguistic aspects.

Limitations of the study and prospective. We believe that this work could be of great use to the political decision-makers responsible for the optimisation of the GSSP. Likewise, it could serve as reference point for other decision-makers in organizations dedicated to designing, developing, implementing, and evaluating PYD-focused extracurricular youth sport programmes. However, the positive effects attributed to this programme should be taken with caution. On the one hand, the requirements that the GSSP fulfills for hosting positive developmental contexts and opportunities for PYD are highlighted, without considering what it does not fulfill in the model of Holt et al. (2017). On the other hand, this analysis is based on the study of a sports programme, but its implementation has not been studied. Our next step will therefore be to study the impact of the programme on the ground so that we can complete the evaluation.

## Data Availability

The original contributions presented in this study are included in this article/supplementary material, further inquiries can be directed to the corresponding author.

## Footnotes

1 The province of Gipuzkoa had 726,033 inhabitants in 2021, according to the National Statistics Institute: https://www.ine.es/jaxiT3/Datos.htm?t=31304.
